# Attenuation of Glucose-Induced Myoglobin Glycation and the Formation of Advanced Glycation End Products (AGEs) by (*R*)-α-Lipoic Acid In Vitro

**DOI:** 10.3390/biom8010009

**Published:** 2018-02-08

**Authors:** Hardik Ghelani, Valentina Razmovski-Naumovski, Rajeswara Rao Pragada, Srinivas Nammi

**Affiliations:** 1 School of Science and Health, Western Sydney University, Sydney, NSW 2751, Australia; h.ghelani@westernsydney.edu.au (H.G.); v.naumovski@westernsydney.edu.au (V.R.-N.); 2 National Institute of Complementary Medicine (NICM), Western Sydney University, Sydney, NSW 2751, Australia; 3 South Western Sydney Clinical School, School of Medicine, University of New South Wales, Sydney, NSW 2052, Australia; 4 Department of Pharmacology, College of Pharmaceutical Sciences, Andhra University, Visakhapatnam 530003, Andhra Pradesh, India; profprrau@gmail.com

**Keywords:** (*R*)-α-Lipoic acid, glucose, glycation, myoglobin

## Abstract

High-carbohydrate containing diets have become a precursor to glucose-mediated protein glycation which has been linked to an increase in diabetic and cardiovascular complications. The aim of the present study was to evaluate the protective effect of (*R*)-α-lipoic acid (ALA) against glucose-induced myoglobin glycation and the formation of advanced glycation end products (AGEs) in vitro. Methods: The effect of ALA on myoglobin glycation was determined via the formation of AGEs fluorescence intensity, iron released from the heme moiety of myoglobin and the level of fructosamine. The extent of glycation-induced myoglobin oxidation was measured via the levels of protein carbonyl and thiol. Results: The results showed that the co-incubation of ALA (1, 2 and 4 mM) with myoglobin (1 mg/mL) and glucose (1 M) significantly decreased the levels of fructosamine, which is directly associated with the decrease in the formation of AGEs. Furthermore, ALA significantly reduced the release of free iron from myoglobin which is attributed to the protection of myoglobin from glucose-induced glycation. The results also demonstrated a significant protective effect of ALA on myoglobin from oxidative damage, as seen from the decreased protein carbonyls and increased protein thiols. Conclusion: The anti-glycation properties of ALA suggest that ALA supplementation may be beneficial in the prevention of AGEs-mediated diabetic and cardiovascular complications.

## 1. Introduction

Myoglobin is an iron-containing protein expressed in the cardiac myocardium and in skeletal muscle and plays an important role in the storage and transport of molecular oxygen for cellular respiration [1,2,3,4,5]. Myoglobin acts as an intracellular nitric oxide (NO) scavenger, thereby protecting mitochondrial respiration [6,7,8]. Due to the enhanced glucose uptake via glucose transporter 1 (GLUT1) in chronic hyperglycaemia, the amino group of myoglobin readily undergoes a non-enzymatic reaction which results in structural and functional changes of the myoglobin [9,10].

The prolonged incubation of myoglobin with glucose produces fructosamine, followed by the formation of advanced glycation end-products (AGEs), which eventually lead to intra- and/or inter-molecular cross-linking of long-lived proteins [11,12,13]. It is known that glucose-mediated glycation also induces oxidative modification of myoglobin by generating protein carbonyl compounds which may be associated with oxidative stress [9,14]. Thus, the increased production of AGEs associated with diabetes is commonly reported as a central cause in the development of diabetic microvascular and macrovascular complications [15] including cardiovascular abnormalities, diabetic ketoacidosis and renal failure [16,17,18,19]. Thus, the inhibition of AGEs-mediated tissue damage and oxidative stress may offer therapeutic potential for preventing or delaying the onset and/or progression of diabetic complications [20].

Although many compounds have been evaluated in vivo and in vitro, no single compound effectively suppresses protein glycation in a clinical setting. Aminoguanidine has been shown to be a potent inhibitor of the protein glycation process and fluorescent AGEs formation in animals and in humans [21]. However, its clinical use is limited due to severe adverse effects such as flu-like symptoms, gastrointestinal problems and anaemia [22,23,24]. Phenyl thiazolium bromide reversed protein cross-linking caused by AGEs in rats [25]. However, it is unstable in physiological buffers. Therefore, there is an urgent need to evaluate new compounds that inhibit protein glycation and thus may be beneficial in preventing diseases mediated by AGEs.

(*R*)-α-Lipoic acid (ALA; 1,2-dithiolane-3-pentanoic acid), also known as thioctic acid, is the biologically active form that is endogenously produced by the body and is recognised as an essential co-factor in mitochondrial respiratory enzymes that catalyse the oxidative decarboxylation reactions [26]. At the cellular level, ALA is reduced to dihydrolipoic acid (DHLA), which has a number of cellular actions including free radical scavenging and modulating oxidative stress and inflammatory pathways [27]. When exogenously administered, ALA is readily absorbed from the gut and has been clinically used in Europe for the treatment of diabetic polyneuropathy [28].

The effect of ALA on protein glycation and AGEs formation has been investigated both in vitro and in vivo [29,30]. Dietary supplementation of ALA in rats chronically fed glucose significantly decreased mitochondrial superoxide in the heart and AGEs formation in the aorta [31]. The chronic supplementation of ALA in fructose-fed rats significantly attenuated AGEs-mediated skin-collagen cross-linking and other physicochemical abnormalities [32]; markedly lowered the circulation of glucose, glycated protein, glycated haemoglobin and fructosamine and prevented the glycation and accumulation of AGEs in isolated rat diaphragm [33]. In obese Zucker rats, the chronic treatment of ALA significantly inhibited protein carbonyls content and improved insulin sensitivity in skeletal muscle [34]. α-Lipoic acid also inhibited AGEs production and down-regulated receptor for advanced glycation end products (RAGE) expression in streptozotocin-induced diabetic rats [35], in human embryonic kidney cells and in rat sensory neurons [36,37]. The topical treatment of ALA nanoparticles significantly down-regulated the expression of RAGE and enhanced cutaneous wound healing in streptozotocin-induced diabetic mice [38].

In an in vitro glycation model containing bovine serum albumin and glucose, ALA markedly inhibited fructosamine, protein carbonyls and fluorescent AGEs production [39,40]. Moreover, ALA markedly suppressed AGEs-induced activation of nuclear factor kappa-light-chain-enhancer of activated B cells (NF-κB) in cultured vascular endothelial cells [41] and in retinal endothelial cells [42]. In another independent study, exogenous administration of ALA diminished AGEs-induced endothelial expression of vascular cell adhesion molecule-1 (VCAM-1) and monocyte binding to endothelium [43]. Furthermore, ALA prevented the up-regulation of AGEs-induced inducible nitric oxide synthase (iNOS) expression and NO production in murine microglial cells [44]. α-Lipoic acid also reduced the AGEs-mediated formation of lipid peroxidation products in human neuronal cells [45,46] and in rat cortical neurones [46]. A more recent investigation in our laboratory has demonstrated that the *R*-enantiomer of ALA significantly inhibited fructose-induced myoglobin glycation and AGEs formation in an in vitro model [47]. In the present study, we investigated the effects of ALA on glucose-induced myoglobin glycation and AGEs formation.

## 2. Materials and Methods

### 2.1. Chemicals and Reagents

(*R*)-α-Lipoic acid, myoglobin, nitro blue tetrazolium (NBT), hydroxylamine hydrochloride, ferrozine, dinitrophenylhydrazine (DNPH), guanidine hydrochloride, ethyl acetate, ethanol, trichloroacetic acid (TCA), dimethyl sulfoxide (DMSO), glucose, 5,5′-dithio-bis (2-nitrobenzoic acid) (DTNB), l-cysteine and aminoguanidine (AG) were purchased from Sigma-Aldrich (St. Louis, MO, USA). Fructosamine and iron standards were obtained from PM Separations (Capalaba DC, QLD, Australia). All other chemicals and reagents were of analytical grade.

### 2.2. Evaluation of the Inhibitory Effect of α-Lipoic Acid on Myoglobin Glycation under Glucose Overload In Vitro

Myoglobin glycation was performed according to the methods previously described by Roy et al. with minor modification [9,13]. Briefly, 500 µL of myoglobin (final concentration: 1 mg/mL) was incubated with 400 µL of glucose (final concentration: 1M) solution at 37 °C in the dark for up to 30 days in the presence or absence of ALA (100 µL; dissolved in DMSO) at a final concentration of 1, 2 and 4 mM. Aminoguanidine (100 µL; dissolved in DMSO), at a final concentration of 5 mM was used as the positive control. After the specified incubation period (10, 20 or 30 days), aliquots of the glycated reaction mixtures were assayed for fluorescent AGEs, free iron, fructosamine (glycated protein), protein carbonyls and protein thiols.

#### 2.2.1. Determination of Fluorescent AGEs Formation

The formation of fluorescent AGEs in the reaction mixture after 30 days of incubation was measured according to the method of Wrobel et al. [48]. Briefly, to 1 mL of the reaction mixture, 250 µL of TCA (100%) was added. The resulting mixture was vortexed for 60 s and centrifuged in a refrigerated centrifuge (Biofuge Stratos, Thermo Scientific, Waltham, MA, USA) at 14,000 rpm for 4 min. The supernatant was collected in a disposable polystyrene cuvette and fluorescence intensity was read at an excitation wavelength 355 nm and emission wavelength 460 nm using a spectrofluorometer (Wallac 1420 Victor 3V, Perkin Elmer, Hong Kong, China). The percentage inhibition of fluorescent AGEs formation was calculated as follows:

Inhibition of fluorescent AGEs (%) = [(Fluorescence intensity of control − Fluorescence intensity of sample)/Fluorescence intensity of control] × 100

#### 2.2.2. Estimation of Free Iron in Glycated Myoglobin (Ferrozine Test)

The free iron, as a measure of glycation-induced iron release in the reaction mixture after 10, 20 and 30 days of incubation, was estimated according to the method of Panter [49]. Briefly, to 250 μL of the reaction mixture, 250 μL of ice cold TCA (20%) was added and centrifuged in a refrigerated centrifuge at 15,000 rpm for 4 min. To 250 μL of the supernatant, 2.5 mL of iron buffer (1.5% hydroxylamine hydrochloride in acetate buffer, pH 4.5) and 50 μL iron colour reagent (0.85% ferrozine in iron buffer) were added. The resultant mixture was incubated at 37 °C for 30 min and the absorbance was measured at 560 nm using an ultraviolet (UV)-visible spectrophotometer (Ultrospec 2100 Pro, Biochrom). The concentration of liberated free iron was calculated as follows:

Concentration of free iron (μg/dL) = (Absorbance of test/Absorbance of standard) × Concentration of standard (μg/dL)

#### 2.2.3. Estimation of Fructosamine (Glycated Myoglobin)

The concentration of fructosamine, as a measure of glycated protein in the reaction mixture after 10, 20 and 30 days of incubation was measured according to the method of Ohkawara et al. [50]. Briefly, to 250 μL of the reaction mixture, 1 mL of 0.5 mM NBT reagent (in carbonate buffer; pH 10.8) was added in a disposable polystyrene cuvette inside a UV-visible spectrophotometer. The absorbance difference at 540 nm after 10 min and 15 min was used to calculate the formation of fructosamine using the following formula:

Concentration of fructosamine (µM) = (Absorbance of test at 15th min − Absorbance of test at 10th min)/(Absorbance of standard at 15th min − Absorbance of standard at 10th min) × Concentration of standard (µM)

#### 2.2.4. Estimation of Protein Carbonyls Content

The level of protein carbonyls, as a measurement of glycation-induced protein oxidation in the reaction mixture after 10, 20 and 30 days of incubation was estimated according to the method of Levine et al. [51]. Briefly, to 200 μL of the reaction mixture, 200 μL of 10 mM DNPH (in 2.5 M hydrochloric acid) was added. After thorough mixing, 250 μL of TCA (30%) was added and centrifuged in a refrigerated centrifuge at 15,000 rpm for 4 min. The pellet was collected and washed three times with 1 mL ethanol: ethyl acetate (1:1) mixture to remove any unreacted DNPH. The pellet was then dissolved in 1 mL of 6 M guanidine hydrochloride (in 20 mM phosphate buffer, pH 6.6), incubated at 37 °C for 15 min and centrifuged in a refrigerated centrifuge at 15,000 rpm for 4 min. The absorbance of the supernatant was then measured at 375 nm using a UV-visible spectrophotometer. The concentration of protein carbonyls was expressed as nanomoles of carbonyls per milligram of protein using the molar absorption coefficient of DNPH (22,000 M^−1^ cm^−1^). 

#### 2.2.5. Estimation of Free Protein Thiols

The concentration of free protein thiols, as a measure of glycation-induced antioxidant defence in the reaction mixture after 10, 20 and 30 days of incubation was measured by Ellman’s assay [52] with minor modifications. Briefly, 70 μL of the reaction mixture was incubated with 130 μL of 5 mM DTNB (in 0.1 M phosphate buffered saline) at room temperature for 15 min and the absorbance was measured at 412 nm using a UV-visible spectrophotometer. The concentration of protein thiols was calculated using a standard curve of l-cysteine and expressed as nanomoles of l-cysteine per milligram of protein.

#### 2.2.6. Statistical Analysis

The results were expressed as a mean ± standard error of the mean (SEM) (*n* = 6). To examine the quantitative differences among the experimental groups, the respective data were subjected to one-way analysis of variance (ANOVA) using GraphPad Prism-5.0 (GraphPad Software Inc., La Jolla, CA, USA) statistical programme. Post hoc comparisons were made using Dunnett’s multiple comparison test. Statistical differences in individual groups at different time points were detected using Student’s paired *t*-test. In all tests, *p* value < 0.05 was used as the criterion for statistical significance.

## 3. Results

### 3.1. Effect of α-Lipoic Acid on the Formation of Fluorescent AGEs

**Figure 1:**
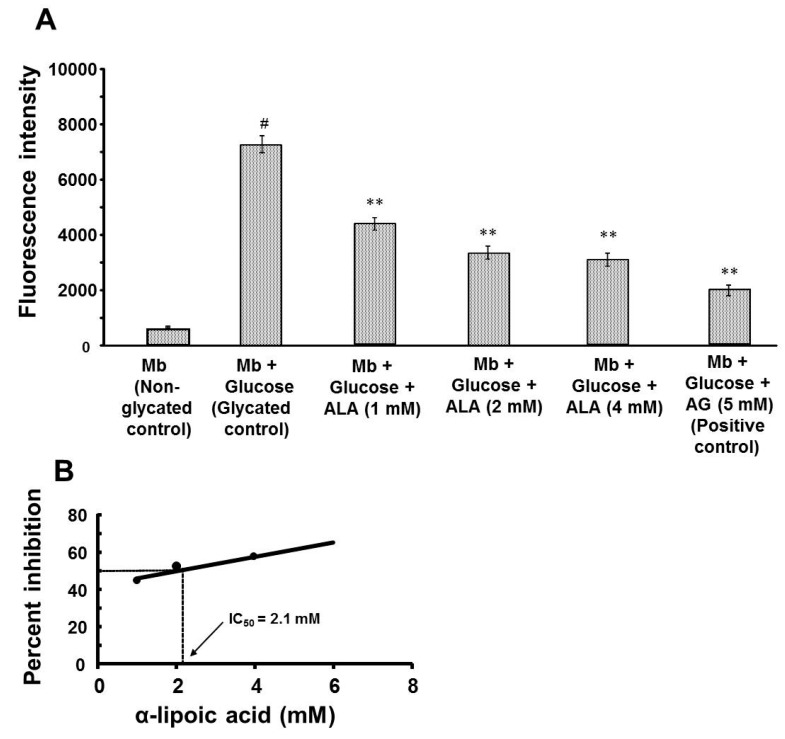
Effect of α-lipoic acid on the formation of fluorescent advanced glycation end products (AGEs) in glucose-induced myoglobin glycation. (**A**) Fluorescence intensity of myoglobin-glucose glycation system alone or after co-incubation with α-lipoic acid or aminoguanidine; and (**B**) The concentration of α-lipoic acid required to inhibit 50% of fluorescent AGEs as determined from linear regression analysis. Each bar represents the mean ± standard error of the mean (SEM) in six separate experiments (*n* = 6); Significant difference from glycated control: ** *p* value < 0.01; Significant difference from non-glycated control: ^#^ *p* value < 0.001; ALA = (*R*)-α-Lipoic acid; AG = Aminoguanidine; Mb = Myoglobin; IC_50_ = concentration required to inhibit 50% of fluorescent AGEs.

The effect of ALA on the formation of fluorescent AGEs in myoglobin-glucose glycation system was observed on day-30 of incubation. As shown in Figure 1A, incubation of myoglobin with glucose (glycated control) significantly (*p* value < 0.001) increased the formation of fluorescent AGEs by 14-fold as shown by increased fluorescence intensity (7633.3 ± 332.5 vs. 539.7 ± 4.8) compared with myoglobin incubation alone (non-glycated control). α-Lipoic acid co-treatment in the myoglobin-glucose glycation system at 1, 2 and 4 mM elicited significant (*p* value < 0.01) concentration-dependent inhibition of the formation of fluorescent AGEs, with a maximum reduction of 56.3% (3333.3 ± 332.5 vs. 7633.3 ± 332.5) at 4 mM concentration compared with the glycated control. The concentration of ALA required to inhibit 50% (IC_50_) of fluorescent AGEs as determined from linear regression analysis was found to be 2.1 mM (Figure 1B). In comparison, the positive control, aminoguanidine (5 mM; a known inhibitor of the glycation process) produced a significant (*p* value < 0.01) 71.6% inhibition of fluorescent AGEs formation (2166.7 ± 147.2 vs. 7633.3 ± 332.5) compared with the glycated control.

### 3.2. Effect of α-Lipoic Acid on Free Iron Release

Table 1 shows the effect of ALA on glycation-induced iron release in the myoglobin-glucose glycation system as observed on day-10, 20 and 30 of incubation. A significant (*p* value < 0.001) 12-fold increase in free iron (117.3 ± 0.4 vs. 9.4 ± 1.4) was observed on day-10 when myoglobin was co-incubated with glucose (glycated control) compared with myoglobin incubation alone (non-glycated control) and moreover, this difference was consistent throughout the study period. Nevertheless, no significant time-dependent change in free iron level was observed in the myoglobin-glucose co-incubation on day-20 or day-30 compared with day-10 value. However, the co-treatment of ALA at 1, 2 and 4 mM concentrations significantly (*p* value < 0.05 to *p* value < 0.01) displayed a concentration-dependent reduction in free iron levels on day-10 compared with the glycated control and moreover, this difference was consistent throughout the study period. On day-30 of co-treatment, ALA significantly (*p* value < 0.01) decreased the free iron levels, with a maximum reduction of 45.1% (67.1 ± 0.2 vs. 122.0 ± 0.5) at the concentration of 4 mM compared with the glycated control. Nevertheless, at all the studied ALA concentrations, no significant time-dependent change in free iron levels was observed within groups on day-20 or day-30 compared with day-10 values. The concentration of ALA required to inhibit 50% (IC_50_) of free iron release as determined from the linear regression analysis was found to be 4.3 mM (Table 1). In comparison, the co-treatment of aminoguanidine (5 mM) produced a significant (*p* value < 0.01) reduction in free iron release on day-10 compared with the glycated control; this was consistent throughout the study period and achieved a maximum reduction of 48.2% (63.2 ± 0.2 vs. 122.0 ± 0.5) on day-30.

### 3.3. Effect of α-Lipoic Acid on Fructosamine Formation

The effect of ALA on fructosamine formation in the myoglobin-glucose glycation system as observed on day-10, 20 and 30 of incubation is shown in Table 2. A significant 1.6-fold increase in fructosamine (759.5 ± 21.6 vs. 486.4 ± 22.7; *p* value < 0.05) was observed on day-10 when myoglobin was co-incubated with glucose (glycated control) compared with myoglobin incubation alone (non-glycated control) and moreover, this difference was more pronounced after day-20 (1131.8 ± 34.6 vs. 557.4 ± 13.5; *p* value < 0.01) and day-30 (1376.9 ± 27.8 vs. 564.9 ± 30.3; *p* value < 0.001) of the study. Moreover, a marked time-dependent increase in fructosamine was observed in myoglobin-glucose co-incubation that gained significance by day-20 (1131.8 ± 34.6 vs. 759.52 ± 21.6; *p* value < 0.05) and day-30 (1376.9 ± 27.8 vs. 759.52 ± 21.6; *p* value < 0.01) compared with day-10. On the other hand, ALA co-treatment at the 1, 2 and 4 mM concentrations significantly (*p* value < 0.05 to *p* value < 0.01) displayed a concentration-dependent reduction in fructosamine levels on day-10 compared with the glycated control and moreover, this difference was consistent throughout the study period. On day-30 of co-treatment, ALA showed a significant (*p* value < 0.01) decrease in fructosamine levels with a maximum reduction of 36.6% (871.8 ± 19.8 vs. 1376.9 ± 27.8) at 4 mM concentration compared with the glycated control. Nevertheless, at all the studied ALA concentrations, no significant time-dependent change in fructosamine levels was observed on day-20 or day-30 compared with day-10 values. The concentration of ALA required to inhibit 50% (IC_50_) of fructosamine as determined from linear regression analysis was found to be 6.2 mM (Table 2). In comparison, the co-treatment of aminoguanidine (5 mM) produced a significant (*p* value < 0.01) reduction in fructosamine on day-10 compared with the glycated control which was consistent throughout the study period achieving a maximum 55.9% reduction (606.1 ± 24.3 vs. 1376.9 ± 27.8) on day-30.

### 3.4. Effect of α-Lipoic Acid on Protein Carbonyls Formation

Table 3 depicts the effect of ALA on protein carbonyls formation in the myoglobin-glucose glycation system as observed on day-10, 20 and 30 of incubation. A significant 2.7-fold increase in protein carbonyls (5.4 ± 0.1 vs. 2.0 ± 0.2; *p* value < 0.001) was observed on day-10 when myoglobin was co-incubated with glucose (glycated control) compared with myoglobin incubation alone (non-glycated control) and moreover, this difference was more pronounced after day-20 (6.8 ± 0.2 vs. 2.3 ± 0.2; *p* value < 0.001) and day-30 (8.2 ± 0.2 vs. 2.3 ± 0.2; *p* value < 0.001) of the study. Furthermore, a marked time-dependent increase in protein carbonyls was observed in myoglobin-glucose co-incubation that gained significance by day-30 (8.2 ± 0.2 vs. 5.4 ± 0.1; *p* value < 0.05) compared with day-10. On the other hand, ALA co-treatment in myoglobin-glucose glycation system at 1, 2 and 4 mM concentrations significantly (*p* value < 0.05 to *p* value < 0.01) displayed a concentration-dependent reduction in protein carbonyls levels on day-10 compared with the glycated control and moreover, this difference was consistent throughout the study period. On day-30 of co-treatment, ALA showed a significant (*p* value < 0.01) decrease in protein carbonyls levels with a maximum reduction of 52.6% (4.6 ± 0.1 vs. 8.2 ± 0.2) at a concentration of 4 mM compared with the glycated control. Nevertheless, at all the studied ALA concentrations, no significant time-dependent change in protein carbonyls levels was observed on day-20 or day-30 compared with day-10 values. The concentration of ALA required to inhibit 50% (IC_50_) of protein carbonyls as determined from linear regression analysis was found to be 3.4 mM (Table 3). In comparison, co-treatment of aminoguanidine (5 mM) produced a significant (*p* value < 0.01) reduction in protein carbonyls on day-10 compared with the glycated control which was consistent throughout the study period achieving a maximum 60.6% reduction (3.1 ± 0.2 vs. 8.2 ± 0.2) on day-30.

### 3.5. Effect of α-Lipoic Acid on Protein Thiols Oxidation

The effect of ALA on free protein thiols in myoglobin-glucose glycation system as observed on day-10, 20 and 30 of incubation is depicted in Table 4. A significant 2.5-fold decrease in free protein thiols (2.8 ± 0.2 vs. 7.2 ± 0.8; *p* value < 0.001) was observed on day-10 when myoglobin was co-incubated with glucose (glycated control) compared with myoglobin incubation alone (non-glycated control) and moreover, this difference was consistent throughout the study period. Furthermore, a marked time-dependent decrease in protein thiol was observed in myoglobin-glucose co-incubation that gained significance by day-30 (1.9 ± 0.2 vs. 2.8 ± 0.2; *p* value < 0.05). On the other hand, ALA co-treatment in myoglobin-glucose glycation system at 1, 2 and 4 mM concentrations significantly (*p* value < 0.05 to *p* value < 0.01) displayed a concentration-dependent increase in free protein thiols levels on day-20 compared with the glycated control and moreover, this difference was consistent throughout the study period. On day-30 of co-treatment, ALA showed a significant (*p* value < 0.01) increase in free protein thiols levels with a maximum increment of 54.7% (3.0 ± 0.2 vs. 1.9 ± 0.2) at 4 mM concentration compared with the glycated control. Nevertheless, at all the studied ALA concentrations, no significant time-dependent change in free protein thiols levels was observed on day-20 or day-30 compared with day-10 values. The concentration of ALA required to increase 50% (EC_50_) of free protein thiols as determined from linear regression analysis was found to be 3.4 mM (Table 4). In comparison, co-treatment of aminoguanidine (5 mM) produced a significant (*p* value < 0.01) increase in free protein thiols on day-10 compared with the glycated control which was consistent throughout the study period achieving a maximum 76.1% increase (3.4 ± 0.5 vs. 1.9 ± 0.2) on day-30.

## 4. Discussion

Various studies have indicated that dietary supplementation with nutrients possessing both antiglycation and antioxidant properties may be a safe complement to traditional therapies aimed at preventing diabetic complications [53,54,55]. In this study, we examined the preventive effects of ALA in glucose-induced myoglobin glycation and AGEs formation using an in vitro glycation model. The observed IC_50_/EC_50_ values of ALA in various anti-glycation assays are depicted in Table 5. The selected concentrations of ALA used in this study were rationalised based on our previous experience and on the published literature [56,57,58]. The clinical pharmacokinetic characteristics of ALA (200 mg/day to 600 mg/day) have been widely reported with varied pharmacokinetic profiles in multiple studies as reviewed by Shay and colleagues [27]. The disparity in the observed oral bioavailability and peak plasma concentration has been attributed to whether the *R*-enantiomer, its salt form, or a racemic mixture of ALA has been used in the studies, plus any associated formulation and/or biopharmaceutical factors. To our knowledge, this is the first study which investigates the protective effects of α-lipoic acid in glucose-induced myoglobin glycation.

Based on fluorescence property, we examined the influence of ALA on the formation of total AGEs. Our results demonstrated that ALA efficiently inhibited glucose-induced AGEs formation which supports our recent paper [47] and other previously published reports on inhibitory effects of ALA on fluorescent AGEs formation [34,36,59]. The possible mechanism of action of ALA in inhibiting the formation of fluorescent AGEs include: (i) blocking the amino groups of protein, thus preventing its glycation with free sugar; (ii) blocking the carbonyl groups of reducing sugars; (iii) preventing the formation of Amadori products by blocking the Schiff’s base to Amadori products conversion; (iv) blocking the Amadori products and dicarbonyl intermediates which may reduce glycation, as well as AGEs formation; and/or (v) preventing autoxidation of glucose and glycoxidation of Amadori products. The protein glycation reaction generates various fluorescent AGEs such as pentosidine and crossline which are implicated in the development of diabetic cardiovascular complications [60,61]. It has been demonstrated that serum fluorescent AGEs (such as pentosidine) were significantly higher in diabetic patients and was associated with an increased incidence of cardiovascular disease [62,63]. Moreover, AGEs can cross-link with extracellular matrix proteins such as collagen, thereby increasing arterial wall and myocardial stiffness. This leads to systolic and diastolic dysfunction of the heart and potentiates heart failure in diabetic patients [64]. Furthermore, binding AGEs to its receptor (RAGE) cause modifications to low-density lipoprotein (LDL) (i.e., oxidation of LDL) and subsequently, generate foam cells, which are hallmarks of atherosclerosis [65]. Therefore, preventing the formation of AGEs or removing cross-linked AGEs is an efficient way of interrupting the glycation cascade and preventing the potential pathological consequences of AGEs.

In our study, ALA displayed a significant, time-dependent inhibition of the formation of fructosamine. A possible mechanism for inhibiting the formation of Amadori products could be either by competing with sugar molecules or protecting the protein amino group from the nucleophilic addition of the carbonyl group of the sugar. In the early stages of protein glycation, the reaction between the carbonyl group of sugar and the amino group of protein form freely reversible Schiff’s base which further rearranges to form a more stable ketoamine or Amadori products such as fructosamine [66]. At this stage, reducing sugar itself undergoes autoxidation in the presence of transition metals and generates various highly reactive superoxide radical and hydroxyl radical. The harmful radicals further accelerate the glycation process to form AGEs. In addition, the Amadori products also react with free protein and generate AGEs [65]. Thus, the reduction of fructosamine would be beneficial in the suppression of AGEs formation and therapeutically delay the occurrence of AGEs-mediated complications.

Furthermore, myoglobin-glucose glycation effectively released free iron from the heme moiety of myoglobin in a time-dependent manner and this was effectively suppressed by ALA. During myoglobin glycation, iron is liberated from the heme and ligated probably to distal histidine in the heme pocket of myoglobin. This iron termed as “mobile reactive iron” can catalyse Haber-Weiss reaction producing free radicals (particularly hydroxyl (OH) radicals), which increase cellular oxidative stress and damage different cellular constituents [12,13,67]. Roy and co-workers demonstrated that in vitro non-enzymatic glycation of myoglobin induces the release of free iron from the heme pocket of myoglobin and the iron release was found to be proportional to the extent of myoglobin glycation [9].

We also examined the influence of ALA against glucose-mediated non-enzymatic glycation and oxidation-dependent damage to myoglobin. In this study, ALA suppressed the formation of protein carbonyls content and oxidation of thiols. ALA is a potent biological antioxidant and is capable of scavenging many free radicals such as hydroxyl groups [68,69]. During protein glycation, reactive di-carbonyl intermediates and protein carbonyl derivatives generate AGEs formation and modify protein structure which is prone to oxidative reaction with amino acids such as cysteine, particularly the thiol side chain. The reactive oxygen species and reactive nitrogen species are also generated during glycation and glycoxidation. These free radicals can oxidize the amino acid side chains of the protein to form a carbonyl derivative and diminish the protein’s oxidative defence by eliminating the thiol groups [70,71]. These alterations are reflective of oxidative protein damage, with oxidative stress and the formation of AGEs. Therefore, a possible mechanism of ALA in suppressing the formation of protein dicarbonyls is scavenging the highly reactive free radicals generated during chronic glycation.

## 5. Conclusions

These findings demonstrate that ALA protects against glucose-mediated myoglobin glycation in vitro by inhibiting the early and intermediate glycation reactions involved in the formation of AGEs. Further studies are warranted to investigate the ability of ALA on late-stage glycation events that lead to the production of AGEs, including AGEs-mediated protein cross-linking and cellular signalling pathways, involved in diabetic and cardiovascular complications.

## Figures and Tables

**Table 1:** Effect of α-lipoic acid on free iron release in glucose-induced myoglobin glycation.

Experimental Group	Concentration of Free Iron (µg/dL)			Percent Inhibition of Free Iron Release	IC_50_ (Day-30)
	Day-10	Day-20	Day-30		
Mb (non-glycated control)	9.4 ± 1.4	10.5 ± 1.3	11.5 ± 1.3	-	4.3 mM
Mb + Glu (glycated control)	117.3 ± 0.4 ^###^	122.0 ± 0.4 ^###^	122.0 ± 0.5 ^###^	-	
Mb + Glu + ALA (1 mM)	92.4 ± 0.3 *	95.2 ± 0.3 **	95.3 ± 0.3 **	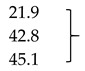	
Mb + Glu + ALA (2 mM)	68.4 ± 0.4 **	70.3 ± 0.6 **	69.8 ± 0.4 **		
Mb + Glu + ALA (4 mM)	65.9 ± 0.5 **	66.7 ± 0.9 **	67.1 ± 0.2 **		
Mb + Glu + AG (5 mM; positive control)	59.1 ± 0.5 **	61.0 ± 0.1 **	63.2 ± 2.0 **	48.2	

Each value represents the mean ± SEM of six (*n* = 6) experiments. Significant difference compared to glycated control at identical times: * *p* value < 0.05; ** *p* value < 0.01; Significant difference from non-glycated control: ^###^ *p* value < 0.001.

**Table 2:** Effect of α-lipoic acid on fructosamine formation in glucose-induced myoglobin glycation.

Experimental Group	Fructosamine (µmol/L)			Percent Inhibition of Fructosamine Formation	IC_50_ (Day-30)
	Day-10	Day-20	Day-30		
Mb (non-glycated control)	486.4 ± 22.7	557.4 ± 13.5	564.9 ± 30.3	-	6.2 mM
Mb + Glu (glycated control)	759.5 ± 21.6 ^#^	1131.8 ± 34.6 ^§,##^	1376.9 ± 27.8 ^§§,###^	-	
Mb + Glu + ALA (1 mM)	742.7 ± 18.2	959.7 ± 40.1 *	1107.5 ± 13.1 **	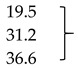	
Mb + Glu + ALA (2 mM)	617.3 ± 16.9 *	795.0 ± 36.1 **	984.0 ± 11.3 **		
Mb + Glu + ALA (4 mM)	501.3 ± 28.3 **	632.3 ± 71.1 **	871.8 ± 19.8 **		
Mb + Glu + AG (5 mM; positive control)	445.2 ± 13.2 **	493.9 ± 21.4 **	606.1 ± 24.3 **	55.9	

Each bar represents the mean ± SEM of six (*n* = 6) experiments; Significant difference compared to glycated control at identical times: * *p* value < 0.05; ** *p* value < 0.01; Significant difference from non-glycated control at identical times: ^#^ *p* value < 0.05; ^##^ *p* value < 0.001; ^###^ *p* value < 0.001; Significant difference from the respective Day-10 value: ^§^ *p* value < 0.05; ^§§^ *p* value < 0.01.

**Table 3:** Effect of α-lipoic acid on protein carbonyls content in glucose-induced myoglobin glycation.

Experimental Group	Protein Carbonyls Content (nmol/mg Protein)			Percent Inhibition of Protein Carbonyls Formation	IC_50_ (Day-30)
	Day-10	Day-20	Day-30		
Mb (non-glycated control)	2.0 ± 0.4	2.3 ± 0.2	2.3 ± 0.2	-	3.4 mM
Mb + Glu (glycated control)	5.4 ± 0.1 ^###^	6.8 ± 0.3 ^###^	8.2 ± 0.2 ^§,###^	-	
Mb + Glu + ALA (1 mM)	4.5 ± 0.1 *	5.0. ± 0.1 *	5.7 ± 0.1 **	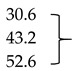	
Mb + Glu + ALA (2 mM)	3.9 ± 0.1 **	4.1 ± 0.2 **	4.4 ± 0.2 **		
Mb + Glu + ALA (4 mM)	3.0 ± 0.1 **	3.2 ± 0.3 **	4.6 ± 0.1 **		
Mb + Glu + AG (5 mM; positive control)	2.9 ± 0.1 **	3.0 ± 0.1 **	3.1 ± 0.2 **	60.6	

Each value represents the mean ± SEM of six (*n* = 6) experiments. Significant difference compared to glycated control at identical times: * *p* value < 0.05; ** *p* value < 0.01; Significant difference from non-glycated control: ^###^ *p* value < 0.001; Significant difference from the respective Day-10 value: ^§^ *p* value < 0.05.

**Table 4:** Effect of α-lipoic acid on protein thiols oxidation in glucose-induced myoglobin glycation.

Experimental Group	Free Protein Thiols (nmol/mg Protein)			Percent Increase of Protein Thiols	EC_50_ (Day-30)
	Day-10	Day-20	Day-30		
Mb (non-glycated control)	7.2 ± 0.4	6.9 ± 0.6	6.7 ± 0.3	-	3.4 mM
Mb + Glu (glycated control)	2.8 ± 0.2 ^###^	2.2 ± 0.1 ^###^	1.9 ± 0.2 ^§,###^	-	
Mb + Glu + ALA (1 mM)	3.1 ± 0.7	2.7 ± 0.1 *	2.5 ± 0.1 **	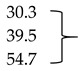	
Mb + Glu + ALA (2 mM)	3.2 ± 0.4	2.8 ± 0.6 *	2.7 ± 0.5 **		
Mb + Glu + ALA (4 mM)	3.5 ± 0.5	3.1 ± 0.3 **	3.0 ± 0.2 **		
Mb + Glu + AG (5 mM; positive control)	4.6 ± 0.4 **	3.7 ± 0.1 **	3.4 ± 0.5 **	76.1	

Each value represents the mean ± SEM of six (*n* = 6) experiments. Significant difference compared to glycated control at identical times: * *p* value < 0.05; ** *p* value < 0.01; Significant difference from non-glycated control: ^###^ *p* value < 0.001; Significant difference from the respective Day-10 value: ^§^ *p* value < 0.05.

**Table 5:** IC_50_/EC_50_ values of ALA in various anti-glycation assays.

Anti-Glycation Assays	IC_50_/EC_50_ of ALA (mM)
Inhibition of fluorescent AGEs	2.1
Inhibition of fructosamine assay	6.2
Inhibition of free iron release	4.3
Inhibition of protein carbonyls	3.4
Protection of free protein thiols	3.4

EC_50_: Concentration of ALA required to increase 50% of free protein thiols.
